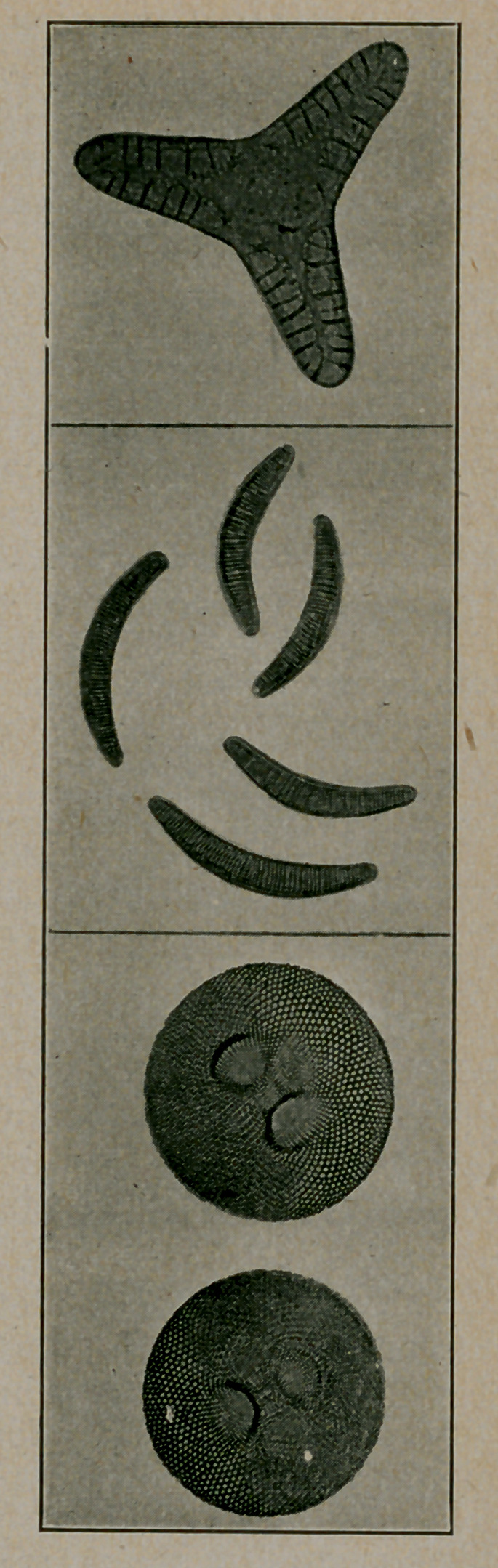# Diatoms

**Published:** 1915-05

**Authors:** 


**Diatoms.** The accompanying cuts of diatoms are supplied by courtesy of the Agassiz Association, ArcAdiA (sic) Sound Beach, Conn., and reproduced from the Guide to Nature. Diatoms are often found in water and, hence, accidentally in stomach, contents, faeces, urine, etc. It is important that physicians familiarize themselves with these adventitious findings and it may be that diatoms cause what is popularly termed sand diarrhoea.

## Figures and Tables

**Figure f1:**
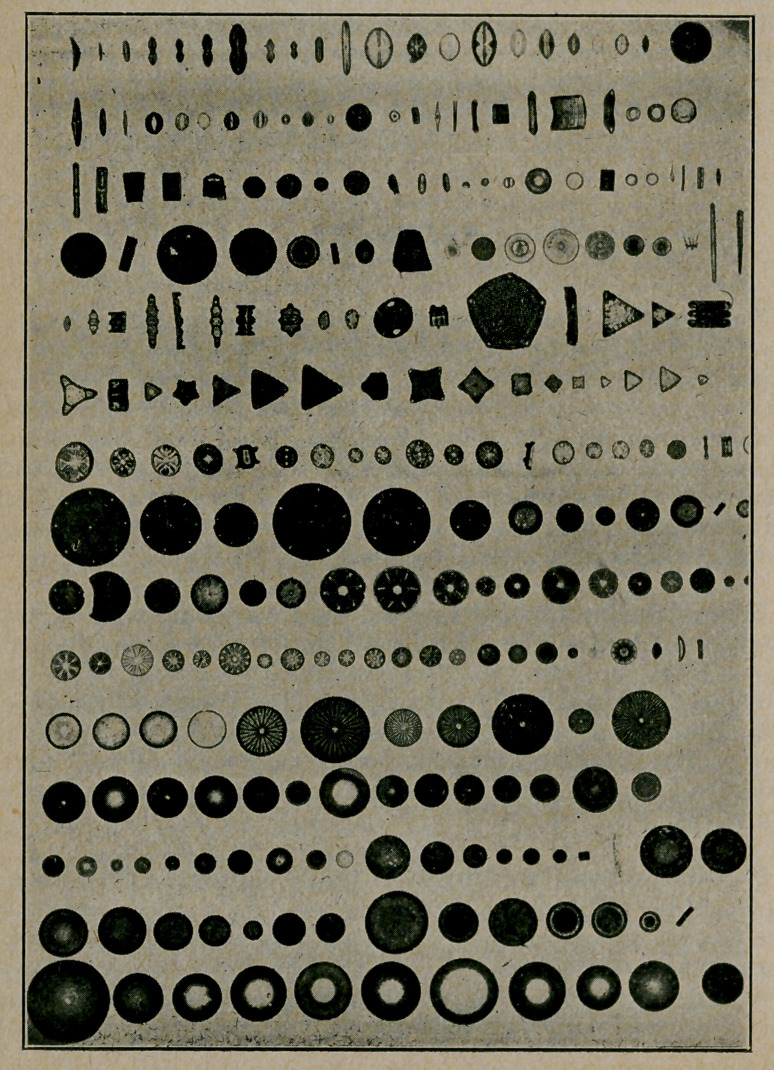


**Figure f2:**
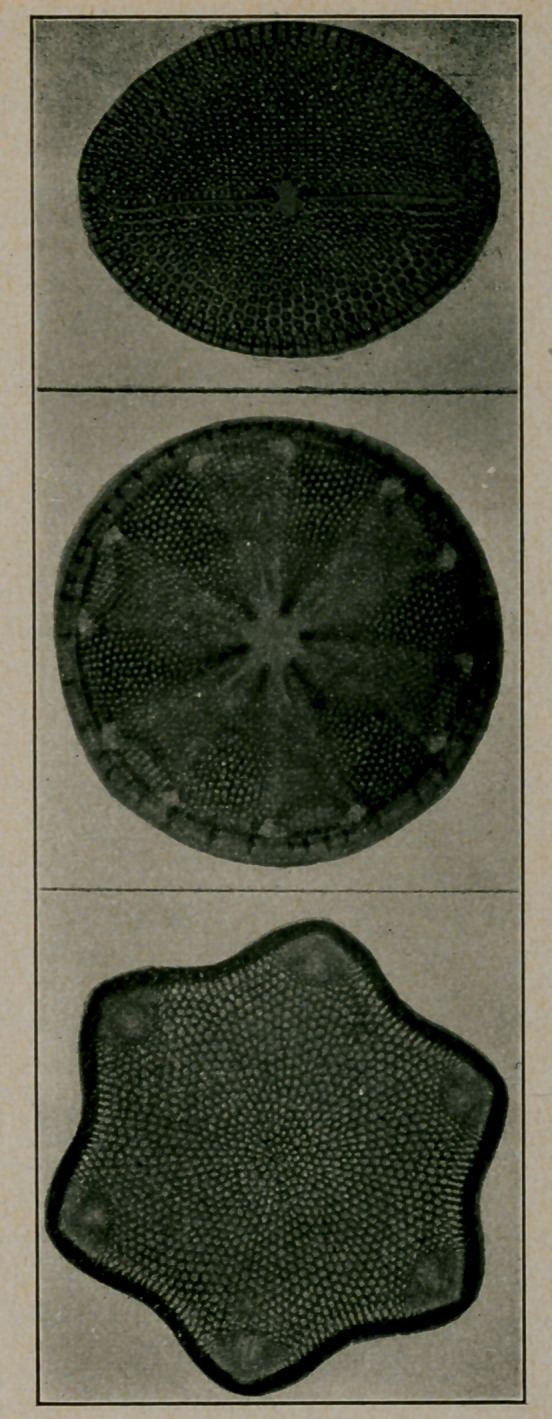


**Figure f3:**